# Dynamic bimodal changes in CpG and non-CpG methylation genome-wide upon CGGBP1 loss-of-function

**DOI:** 10.1186/s13104-018-3516-1

**Published:** 2018-07-02

**Authors:** Divyesh Patel, Manthan Patel, Bengt Westermark, Umashankar Singh

**Affiliations:** 1 HoMeCell Laboratory, Biological Sciences and Engineering, Indian Institute of Technology Gandhinagar, Gujarat, 382355 India; 2 Department of Immunology, Genetics and Pathology, and Science for Life Laboratory, Uppsala University, 75185 Uppsala, Sweden

**Keywords:** Cytosine methylation, CGGBP1, Genome-wide bisulfite sequencing, GC-skew

## Abstract

**Objectives:**

Although CpG methylation is well studied, mechanisms of non-CpG methylation in mammals remains elusive. Studying proteins with non-CpG cytosine methylation-sensitive DNA-binding, such as human CGGBP1, can unveil cytosine methylation regulatory mechanisms. Here we have resequenced a published genome-wide bisulfite sequencing library and analyzed it at base level resolution. CpG, CHG and CHH (where H is any nucleotide other than G) methylation states in non-targeting or CGGBP1-targeting shmiR lentivirus-transduced cells have been analyzed to identify how CGGBP1 regulates CpG and non-CpG methylation.

**Results:**

We report that CGGBP1 acts as a dynamic bimodal balancer of methylation. Both gain and loss of methylation observed upon CGGBP1 depletion were spatially overlapping at annotated functional regions and not identifiable with any sequence motifs but clearly associated with GC-skew. CGGBP1 depletion caused clustered methylation changes in *cis*, upstream of R-loop forming promoters. This was complemented by clustered occurrences of methylation changes in proximity of transcription start sites of known cytosine methylation regulatory genes, altered expression of which can regulate cytosine methylation in *trans*. Despite low coverage, our data provide reliable estimates of the spectrum of methylation changes regulated by CGGBP1 in all cytosine contexts genome-wide through a combination of *cis* and *trans*-acting mechanisms.

**Electronic supplementary material:**

The online version of this article (10.1186/s13104-018-3516-1) contains supplementary material, which is available to authorized users.

## Introduction

Cytosine methylation patterns are established and maintained with specificity at functional locations in our genome. Intricate patterns of cytosine methylation genome-wide are required for gene expression regulation, allele-specific functions of genomic loci, genomic integrity and silencing of repetitive elements. Discovering novel broad spectrum cytosine methylation regulators is of great importance for a holistic understanding of mechanisms of cytosine methylation and their consequences. One such recently reported cytosine methylation regulator protein is CGGBP1 [1].

CGGBP1 regulates retrotransposons, genomic integrity and transcription [2, 3]. Most recently, CGGBP1 has been shown to mitigate cytosine methylation at repetitive regions [1]. Abrogation of CGGBP1 function disturbs CpG methylation patterns, with both, gain and loss of methylation identifiable. While LINE-1 elements exhibit only a gain of methylation, the Alu-SINEs exhibit both increase and decrease in CpG methylation upon CGGBP1 depletion. In the absence of CGGBP1, the hypermethylation of repetitive regions manifests as a net increase in genomic cytosine methylation levels. Despite evidences that CGGBP1 regulates the transcript levels of cytosine methylation regulatory enzymes (both positive regulators such as methyl transferases as well as the negative regulators including the TET family of oxidases) [1] it remains unknown how it regulates cytosine methylation.

Following up from our previous work, here we present a base-level analysis of cytosine methylation change caused by CGGBP1-depletion. To attempt enhancing the mapping efficiency since last report, we resequenced the whole-genome bisulfite-converted DNA libraries described earlier [1]. We find that CGGBP1 depletion causes both loss and gain of cytosine methylation. The specific targets of methylation change by CGGBP1 depletion include regions with a GC-skew. We also show that the abrogation of CGGBP1 function results in altered TSS methylation patterns for cytosine methylation regulatory genes previously shown [1] to be deregulated by CGGBP1 depletion. Giving a mechanistic insight into our previous findings, these results strongly implicate CGGBP1 as a maintainer of CpG and CH methylation patterns both in *cis* and *trans*.

## Main text

New genome-wide bisulfite converted DNA sequence data were obtained from libraries described previously [1] in an attempt to increase read mappability. Normal human foreskin fibroblasts 1064Sk were transduced with CGGBP1-targeting or non-targeting lentiviral shmiRs. CGGBP1 knock-down was confirmed by western blot and genomic DNA was extracted. After bisulfite conversion, Illumina paired-end sequencing library was prepared separately for S1 and S2 with Lambda DNA spike. Reads, acquired as paired were unpaired before mapping to restrict methylation state determination only for actually sequenced segments of the genome (more details in [1] and Additional file 1: Methods section).

A total of 27,587,190 sequencing reads from control shmiR and 21,875,927 reads from CGGBP1 shmiR-treated sample (hereafter referred to as S1 and S2 respectively) were mapped to hg38. Reinforcing our previously published findings, the abundance of G+C was higher in S2 compared to S1 whereas that of A+T was lower in S2 compared to S1 (Additional file 1: Table S1). As shown previously, this unexpected nucleotide composition bias is due to a lower C-to-T change upon bisulfite treatment in CGGBP1-depleted sample due to higher levels of cytosine methylation. Since repetitive sequences including L1-LINEs, Alu-SINEs and tandem repeats undergo gain of CpG methylation upon CGGBP1-depletion [1], the non-mappability of sequence reads at repetitive regions reduces the methylated cytosine counts thereby undermining the magnitude of gain of cytosine methylation.

Indeed by comparing the differences in A+T to C+G shift, we found that the unmapped reads of CGGBP1-depleted samples had a small but highly significant 1.88% higher GC content (in CpG, CHG and CHH contexts all combined) than that of the control sample (Additional file 1: Table S2) which was 0.09% at mapped reads (Additional file 1: Table S2). The strongest defining feature of the unmapped reads was the approximately tenfold higher GC content than mapped reads implying that the unmapped reads were extremely GC-rich and methylated, thus resistant to C-to-T conversion (Additional file 1: Table S2). Collectively, the unmapped reads seem to belong to repeats (hence remaining not uniquely mapped) and have high methyl-cytosine content which further increases (as a net change) upon CGGBP1 depletion in all cytosine contexts.

To further analyze the mapped sequence reads at a base level resolution, we retained only those cytosines that were covered in S1 and S2 sequence data both and classified them as undergoing change of methylation or not.

Out of 173,053,153 uniquely mapped cytosines, 15,587,386 exhibited gain of methylation (GoM) and 16283211 exhibited loss of methylation (LoM) upon CGGBP1 depletion. The remaining exhibited no change of methylation thus remaining unmethylated (RuN) or retained methylation (RoM). A total of 10,398,259 (66.71%) GoM and 11,240,514 (69.03%) LoM cytosines were located in repeats as determined by comparative base counts of sequences fetched from unmasked hg38 against repeat-masked hg38 (Additional file 1: Table S3). These results reinforced that bidirectional methylation changes due to CGGBP1 depletion are more than expected at repetitive DNA.

**Fig. 1:**
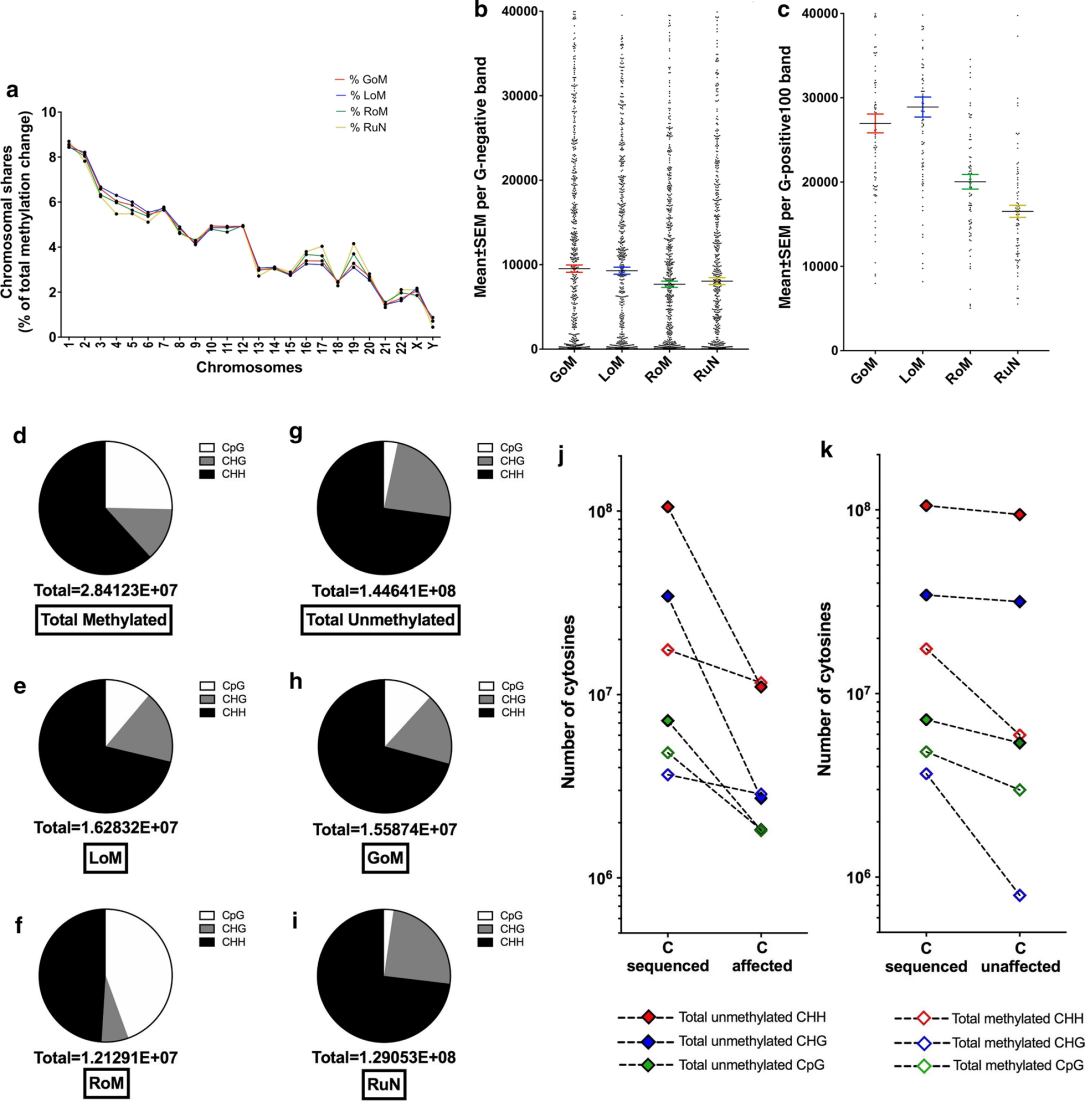
CGGBP1 regulates cytosine methylation in a GC-content and cytosine context dependent manner. **a** Chromosome-wise distribution of cytosines exhibiting methylation changes (GoM and LoM) or no methylation changes (RoM and RuN). The similar distribution of the four methylation change states on all the chromosomes showed no major chromosomal preference for CGGBP1-regulation of cytosine methylation. **b**, **c** Measurement of intra-chromosomal variabilities in methylation states in S1 and S2 shows preference for GoM in G-rich R-bands (Giemsa-negative) and LoM in G-bands (Giemsa positive 100). In R-bands, the paired GoM and LoM events had closely related values like RoM and RuN events but GoM was significantly more than LoM (paired t-test p value = 2.239e−012) (**b**). However, at G-bands the methylation state was reversed and LoM was higher than GoM (p value = 0) (**c**). Similarly, while RuN was significantly higher in R-bands (paired t-test p value = 0) (**b**), RoM was higher in G-bands (paired t-test p value = 0.000636). For paired t-test, n = 780 R bands in B and n = 81 G bands in **c**. **d**–**k** The methylated and unmethylated fractions of cytosines in all three contexts have differential susceptibility to methylation change in absence of CGGBP1 function. **d**–**f** The context-wise distribution of methylated cytosines in S1 (**d**) underwent LoM wherein the proportion of CpG was lower and that of CHG and CHH were more than expected (**e**). From the same pool of cytosines methylated in S1 (**d**) that retained methylation upon CGGBP1 loss-of-function had a highly enriched CpG fraction and lower CHG and CHH fractions (**f**). **g**–**i** The context-wise distribution of unmethylated cytosines in S1 (**g**) that underwent GoM (**h**) also displayed an unexpected and disproportionate increase in CpG context. The context distribution amongst the cytosines that remained unmethylated upon CGGBP1 loss-of-function displayed a reduction in CpG context (**i**). A comparison of **d** and **g** clearly shows that the major fraction of CpG context was methylated in presence of CGGBP1 whereas CHH and CHG together comprised the most of unmethylated fractions. Comparison of **e** and **h** show that despite differences in the absolute numbers as well as relative abundance of the three contexts in methylated and unmethylated pools in S1 (**d**, **g**), the GoM (**e**) and LoM (**h**) cytosines were unexpectedly similar in magnitude with near identical context composition. If LoM and GoM were occurring randomly in the methylated and unmethylated pools of cytosines, then the magnitude and context distributions observed in **d** and **g** were proportionately expected in **e** and **h** respectively. Obs/Exp analyses of **e** and **h** against **d** and **g** revealed a highly significant unexpected composition of **e** and **h** (refer to Additional file 1: Table S6). **j** Plotting of the number of cytosines sequenced in S1 that underwent methylation change upon CGGBP1 depletion shows a disproportionate change in methylation states such that the GoM and LoM are quantitatively coincidental. **k** Conversely to **j**, the number of cytosines sequenced in S1 that resisted methylation change upon CGGBP1 depletion are disproportionately different and non-coincidental. All graphs are generated using GraphPad Prism7

The GoM and LoM cytosines showed an even chromosomal distribution (Fig. 1a) although CGGBP1-binding is more than expected on X chromosome [4] suggesting that DNA-binding and GoM/LoM are not essentially linked processes. An analysis of the presence of GoM and LoM cytosines in the R-bands (GC-rich) and G-bands (GC-poor) genome-wide revealed that CGGBP1 depletion induces GoM in the euchromatic G-negative regions and LoM in heterochromatic G-positive regions (Fig. 1b, c and Additional file 1: Fig. S1A). The cytosine context most amenable to change in methylation upon CGGBP1 depletion was CpG followed by CHH and CHG (Additional file 1: Fig. S1, B–D, Tables S4, S5).

The cytosines methylated in control sample S1 (Fig. 1d) could have two fates, either LoM (Fig. 1e) or RoM (Fig. 1f), in S2. Similarly, the cytosines unmethylated in S1(Fig. 1g) could either exhibit GoM (Fig. 1h) or RuN in S2 (Fig. 1i). Approximately 90% of cytosines covered by more than one read per sample exhibited clear inter-sample variation only. From S1 to S2, different percentages (57.31 and 10.77%) but unexpectedly highly similar numbers of cytosines underwent LoM and GoM respectively (Fig. 1d, e). This unexpected similarity in the number of cytosines was indeed restricted only to GoM and LoM events and not RoM or RuN events (Fig. 1e, h compared with f and i respectively). A Chi square test between expected (sample S1) and observed (sample S2) values of cytosine methylation changes revealed significant difference for all contexts (Additional file 1: Table S6). An overwhelming 90.4% CHG and 85.7% CHH cytosines remained unmethylated whereas in the CpG context only 40% cytosines remained unmethylated as expected (Fig. 1j, k). Such an equivalence between the magnitudes of LoM and GoM (achieved by 57.31 and 10.77% of S1 methylated and unmethylated cytosines respectively) strongly indicated that CGGBP1 depletion simultaneously induces mechanisms that cause GoM and LoM with quantitative convergence (Fig. 1j, k).

By merging (distance and length more 13 bases minimum), 1.09 million GoM and 1.17 million LoM regions were obtained. After filtering of overlapping GoM and LoM regions, only less than 10% of the LoM and GoM regions were retained (73,924 GoM regions and 71,918 LoM regions). These findings suggested that CGGBP1 maintains counteracting mechanisms to ensure no runaway methylation change in any single direction. Interestingly, these regions contained < 1% Alu-SINEs (expected value > 10%) but > 17% L1-LINE (as expected) showing that L1 elements are a consistent target of CGGBP1-dependent methylation regulation (Additional file 1: Table S7).

**Fig. 2:**
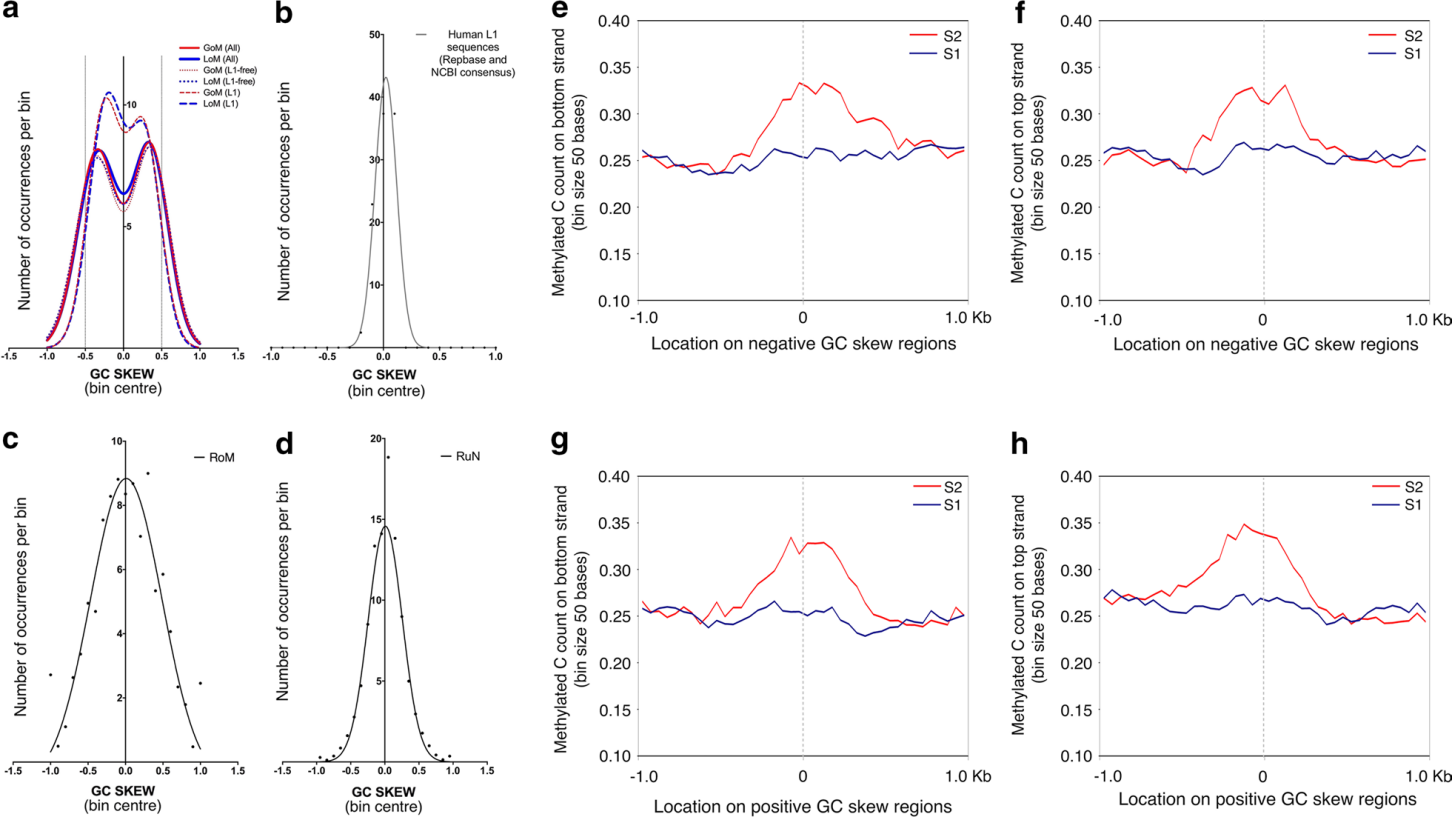
Genomic regions dependent on CGGBP1 for stability of cytosine methylation have inter-strand G/C asymmetry. **a** A frequency plot of GC-skew calculated as {(G−C)/(G+C)} for all GoM (red solid line) and all LoM (blue solid line) regions showed a clear clustering of GoM and LoM regions into two groups; one peaking near − 0.5 and other around + 0.5. The distribution of the data could not be fitted with a single Gaussian curve, but with a sum of two Gaussian curves with very high confidence (Additional file 1: Table S10). When these datasets were split into L1-LINEs (nearly 20% of the GoM and LoM sets; dashed broken lines) and non-L1 regions (dotted lines), we observed a clear difference between the L1 sequences versus the rest with the GoM-L1 and LoM-L1 sequences exhibiting lesser GC-skew than the non-L1 GoM and LoM sequences. However, all of these could be fitted only with a sum of two Gaussian curves. **b** The GC-skew observed with the GoM-L1 and LoM-L1 sequences was unexpected as the L1 sequences from Repbase and NCBI L1 consensus showed an absolutely Gaussian distribution of GC-skew centred near zero. **c**, **d** The RoM and RuN regions did not display the GC-skew as seen for GoM and LoM regions. The GC-skew frequency for RoM and RuN was centred around zero in a binomial fashion. **e**–**h** GC-skew regions genome-wide are prone to methylation gain upon CGGBP1 depletion. The distribution of methylated cytosines centred at the middle of GC-skew regions displayed a binomial increase in methylation on both the strands in the absence of CGGBP1 function. This increase in methylation is highly specific and restricted to less than 1 kb flanks of the GC-skew regions genome-wide [6] with mean length of 747 ± 482 bp. **e** Negative GC-skew, methylation on bottom strand. **f** Negative GC-skew, methylation on top strand. G: Positive GC-skew, methylation on bottom strand. **h** Positive GC-skew, methylation on top strand. Red line = S2, blue line = S1. X axis represents genomic location from the centre of GC-skew regions. Y axis represents methylated cytosine counts in bins with sizes as indicated. Plots were generated using deepTools [8]

Next, we measured the occurrence of repeat-free GoM and LoM regions in genomic landmarks with cytosine methylation-dependent functionality; Enhancers (permissive), TSSs (permissive or robust), Insulators, TADs and LADs. These findings are summarized in Additional file 1: Table S8. The exclusive GoM and LoM regions showed maximum intersects with insulator sequences characterized as CTCF-binding sites [5] (Additional file 1: Table S9). Of all the genomic landmarks examined (Additional file 1: Fig. S2, A–F), most noticeably the insulator sequences showed a central enrichment of methylation events (Additional file 1: Fig. S2A) whereas the permissive enhancer elements showed a reduction in methylation levels at the centre (Additional file 1: Fig. S2B). As positive controls, we did observe a highly specific and strong enrichment of cytosine methylation at LINE-1 elements undergoing GoM (Additional file 1: Fig. S3A) or LoM (Additional file 1: Fig. S3B). In search for sequence features that are associated with methylation regulation by CGGBP1, we measured the inter-strand GC-content asymmetry. The GC-content distribution of GoM and LoM sequences showed an inter-strand skew of GC-content (GC-skew) (Fig. 2a). A frequency plot of GC-skew demonstrated a sum-of-two-Gaussian distribution (peaks at − 0.5 and + 0.5 approximately) of the skew with repeat-free regions exhibiting the highest skew and LINE-1 elements undergoing GoM or LoM showing the least skew (Fig. 2a). LINE1 sequences from RepBase showed no skew (Fig. 2b). No GC-skew was seen in the RoM and RuN sequences (Fig. 2c, d). These results showed that CGGBP1 regulates methylation at genomic regions of inter-strand G/C asymmetry, including a subset of LINE-1 repeats with significantly high GC-skew (Additional file 1: Table S10). Despite strong GC-skew, less than 10% of GoM and LoM sequences were predicted as G4-quadruplexes forming (not shown). However, G4 quadruplex-forming and GC-skew containing replication origins characterized by ORC1, PHIP and ORCA occupancy showed consistently increased methylation levels in S2 (Additional file 1: Table S11 and Fig. S4).

GC-skew regions genome-wide [6] showed an increase in 0.5 kb flanks in S2 (Fig. 2e–h). By plotting methylation signals in S1 and S2 for all the known GC-skew TSSs [6] in a strand-specific manner, we found that the methylation gain in S2 was always in the immediate upstream region relative to the direction of transcription (Additional file 1: Fig. S5). GC-skew regions are also associated with promoters of TSSs [7] that have a strong R-loop formation tendency. Genome-wide R-loop formation has been marked through sequencing of DNA-RNA hybrid regions [6]. 35,664 unique LoM and 29,566 unique GoM regions turned out to contain at least one or more R-loop forming sequences. When we measured the distance of these R-loop containing GoM or LoM regions from TSSs (both robust and permissive separately), we found that there was a specific increase in S2 within 0.5 kb flanks of the TSSs (Additional file 1: Fig. S6). It followed from these emphatic findings that CGGBP1 is potentially a *cis*-regulator of transcription of genes with GC skew TSSs that form R-loops.

**Fig. 3:**
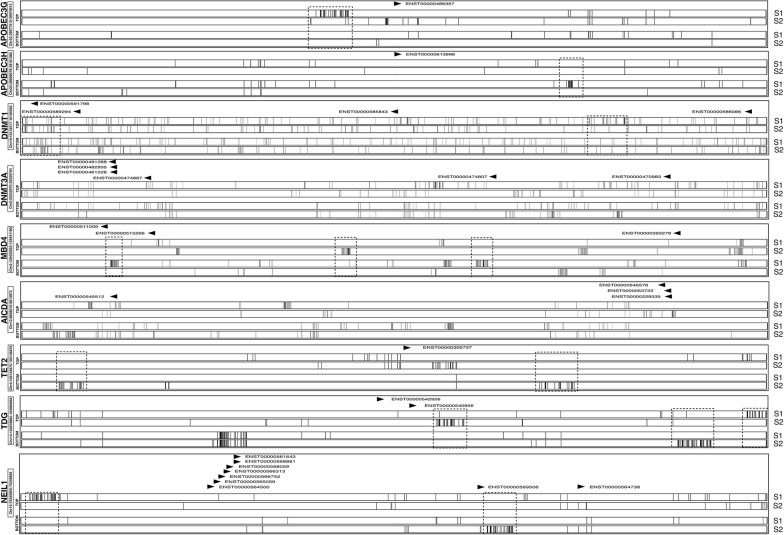
CGGBP1 regulates methylation at TSSs of the cytosine methylation regulatory genes. Cytosine methylation levels in 1 kb flank from TSSs of various transcripts the cytosine methylation regulatory genes (DNMT1, DNMT3A, TET2, AICDA, TDG, NEIL1, MBD4, APOBEC3H and APOBEC3G) was plotted for both the samples S1 and S2 and for both the strands (top and bottom). The cluster of cytosines exhibiting methylation change is highlighted by boxes with dashed lines. For each gene, direction of transcription is marked by arrowheads along-with ENSEMBL transcript ID. All transcripts in the regions are not shown as in Additional file 1: Fig. S7. Plots were generated using deepTools [8] and compiled in Keynote (Apple)

CGGBP1 regulates expression of a subset of genes that regulate cytosine methylation [1]. Of all the known TSSs of these genes, many (for example DNMT1, DNMT3A, TET2, AICDA, TDG, NEIL1, MBD4, APOBEC3H, APOBEC3G, and APOBEC3A) underwent strand-specific methylation changes in response to CGGBP1 depletion (Fig. 3 and Additional file 1: Fig. S7). With these findings we concluded that in addition to the *cis* regulation at GC skew regions, CGGBP1 also regulates cytosine methylation in *trans* through transcription modulation of cytosine methylation establishing and maintenance genes.

To conclude, our findings suggests that CGGBP1 maintains a balanced methylation state for all cytosine contexts. These mechanisms seem to be dual: in *cis* at GC-skew R-loop regions and in *trans* through cytosine methylation regulatory gene promoters. Our results show that CGGBP1 is a methylation-regulatory protein that maintains a balance between cytosine methylation enhancing and mitigating mechanisms independent of the nucleotide sequence and cytosine context. Methylation regulation by CGGBP1 is instead routed through nucleotide composition bias and secondary structure formation ability of the DNA strands, such as R-loops.

## Limitations

The results are derived from re-sequencing of the previously published WGBS libraries and the current analysis is well controlled. However the mapping efficiency and hence the coverage of the sequencing is not very high. Although higher sequencing coverage per cytosine makes such an analysis more robust, it has been a challenge to retain repeats in WGBS analyses and maintain high mappability, especially as CGGBP1 binds to and targets methylation at repetitive sequences. The data shall be viewed in the light of these limitations of working with a repeat-binding protein and inherently low mappability of these sequencing datasets. These findings (based on rigorous computational analyses with proper controls) when read alongside our previously published work shall provide strong evidence for the complex role CGGBP1 plays in cytosine methylation.

## Additional file

**Additional file 1.** A total of Tables S1 to S11 and Figures S1 to S7 with legends, details of methods and additional references are contained in the combined additional data file.

## Abbreviations

CpG: 5′-(C)-p-(G)-3′ dinucleotide
CHG: 5′-(C)-p-(A/T/C)-p-(G)-3′ trinucleotide
CHH: 5′-(C)-p-(A/T/C)-p-(A/T/C)-3′ trinucleotide
CGGBP1: CGG triplet repeat binding protein 1
DNMT1: DNA methyl transferase 1
DNMT3A: DNA methyl transferase 3A
DNMT3B: DNA methyl transferase 3B
DNMTL: DNA methyl transferase L
RNA Pol II: RNA polymerase II
LINE-1: long interspersed nuclear elements
Alu-SINEs: Alu-short interspersed nuclear elements
TET: ten-eleven translocation family protein
TSS: transcription start site
MEME: multiple Em for motif elicitation
fimo: find motif
dreme: discriminative regular expression motif elicitation
QGRS: quadruplex forming G-rich sequences
CTCF: CCCTC-binding factor
GoM: gain of methylation
LoM: loss of methylation
RoM: retention of methylation
RuN: remaining unmethylated
TADs: topologically-associated domains
LADs: lamina-associated domains
ORCA: origin recognition complex subunit 1
ORC1: origin recognition complex subunit 1
PHIP: pleckstrin homology domain interacting protein
TET2: tet methylcytosine dioxygenase 2
AICDA: activation-induced cytidine deaminase
TDG: thymine DNA glycosylase
NEIL1: nei like DNA glycosylase 1
MBD4: methyl-CpG binding domain 4
APOBEC3H: apolipoprotein B mRNA editing enzyme catalytic subunit 3H
APOBEC3G: apolipoprotein B mRNA editing enzyme catalytic subunit 3G
APOBEC3A: apolipoprotein B mRNA editing enzyme catalytic subunit 3A
ES cells: embryonic stem cells
S1: sample 1 (non-targeting control shmiR lentiviral transduced sample)
S2: sample 2 (CGGBP1-targeting shmiR lentiviral transduced sample)
shmiR: short hairpin RNA in micro-RNA backbone
