# Supplementary material for: Dynamic bimodal changes in CpG and non-CpG methylation genome-wide upon CGGBP1 loss-of-function
Source: BMC Res Notes. 2018 Jul 2;11:419. doi: 10.1186/s13104-018-3516-1 (PMC6027561; doi:10.1186/s13104-018-3516-1)
Supplement: Supplementary file 1 — Additional file 1. A total of Tables S1 to S11 and Figures S1 to S7 with legends, details of methods and additional references are contained in the combined additional data file. [file 13104_2018_3516_MOESM1_ESM.pdf]

SUPPLEMENTARY INFORMATION FILE

Dynamic bimodal changes in CpG and non-CpG methylation genome-wide upon CGGBP1 loss-of-function

Divyesh Patel*[1], Manthan Patel*[1], Bengt Westermark[2], Umashankar Singh†[1].

1. HoMeCell Laboratory, Biological Sciences and Engineering, Indian Institute of Technology Gandhinagar, Gujarat 382355, India.
2. Dept of Immunology, Genetics and Pathology, and Science for Life Laboratory, Uppsala University, 75185, Uppsala, Sweden.
* Both authors contributed to this work equally
E-mail address: divyeshkumar.patel@iitgn.ac.in, patel.manthan@iitgn.ac.in, bengt.westermark@igp.uu.se
† Corresponding author: usingh@iitgn.ac.in

Legends for Supplementary Tables

Table S1: Percentage of A+T and G+C in total sequenced data of S1 (in presence of CGGBP1) and S2 (after depletion of CGGBP1).

Table S2: Percentage distribution of A+T and G+C in mapped and unmapped sequences of S1 and S2. For a 2 x 2 contingency analysis (Chi-square test) of A+T and G+C in S1 and S2, the p value < 0.0001 for mapped, unmapped and all combined reads.

Table S3: Distribution of cytosines undergoing change in methylation (GoM or LoM) upon CGGBP1 loss-of-function in repeats (N) and non-repeats (C or G).

Table S4: Context-wise abundance of GoM and LoM (as ratios) of all cytosine sequenced in both the samples.

Table S5: Context-wise abundance of total methylated and total unmethylated cytosines (as ratios) of all cytosine sequenced in both the samples.

Table S6: Chi-square test (using GraphPad Prism 7) for significance for the data presented in figure 1 (D to I) show that the difference between S1 and S2 are highly significant.

Table S7: Repeat content analysis (using RepeatMasker) in consistently differentially methylated sequences between S1 and S2.

Table S8. Pattern of methylation change occurring in different genomic landmarks upon CGGBP1 depletion. Observation summaries presented here are derived out of only those cytosines that were sequenced in both samples S1 and S2 and methylated in at least on of them.

Table S9: Overlap of repeat free GoM and LoM regions with genomic landmarks. This table represents total length and number of overlaps observed for each genomic landmark.

Table S10: Goodness of fit for the sum-of-two Gaussians and Gaussian distributions of GC-skew of GoM and LoM regions presented in figure 2A.

Table S11: Inter- and intra-strand correlation of GoM and LoM at replication origins. Replication origins with no change in methylation in both S1 and S2 have been excluded. Remarkably and unexpectedly, the GoM and LoM events are highly correlated on the same strand as compared to either GoM or LoM on separate strand. This shows that at the replication origins the methylation change in opposite directions tend to be concurrent on the same strand. The values presented are R correlation coefficients. Number of pairs = 5788 minimum and 7741 maximum for ORC1, 5666 minimum and 6704 maximum for ORCA Early, 5211 minimum and 6159 maximum for ORCA Mid, 715 minimum and 806 maximum for ORCA Late, and, 14989 minimum and 12409 maximum for PHIP.

Legends for Supplementary figures

Fig S1: A: Distribution of cytosines either exhibiting change (GoM or LoM) or no change (RoM or RuN) of methylation upon CGGBP1 loss-of-function in Giemsa bands. Higher GoM/LoM ratios were associated with G-rich Giemsa-negative R-bands with progressive decline in GoM/LoM ratio from G-negative towards fully Giemsa- positive G-100 bands. Welch's unpaired T test was performed between the GoM/LoM values for all combinations of G-neg (n=780), G-pos-25 (n=87), G-pos-50 (n=121), G-pos-75 (n=89) and G-pos-100 (n=81). The p values ranged between, maximum 0.0093 and minimum <0.0001. B to D: A base level measurement of cytosine methylation states in the three contexts CpG, CHG and CHH showed a highly context- dependent effect of CGGBP1-depletion on cytosine methylation. In CpG context (B) the normally higher levels of methylated over unmethylated cytosines in S1 was reversed by CGGBP1 depletion. Hence in S2 the net methylation state of CpG was more unmethylated than methylated (S1 total methylation compared to S2 total methylation, Obs/Exp Chi-square p value). C and D: The

unmethylated cytosine count also increased in the CHG (C) and CHH (D) contexts highly significantly (Obs/Exp Chi-square p value < 0.0001 for all contexts). However the balance between unmethylated and methylated cytosines was not reversed by CGGBP1 depletion. This differential effect of CGGBP1 depletion on CpG unlike CHG and CHH contexts could be partially due to the prevalence of the CHG and CHH contexts (the Y axes in C and D are higher by an order of magnitude than in B).

Fig S2: CGGBP1 depletion causes methylation change that is associated with functional genomic regions. A: Cytosine methylation levels in 2.5 Kb flank from midpoint of CTCF-binding sites was plotted. Increased cytosine methylation level was observed at the midpoint of CTCF-binding sites. B: Similarly, cytosine methylation level in 10 Kb flank from midpoint of permissive enhancers was plotted. Decreased cytosine methylation level was observed at the midpoint of the permissive enhancer elements. C and D: Distribution of methylated cytosines in 10 Kb flank from TAD boundaries (TAD start sites (C) and TAD end sites (D)) was plotted. Cytosine methylation level was not altered at TAD boundary in presence and absence of CGGBP1. E and F: Distribution of methylated cytosines in 1 Mb flanks of LAD boundaries (LAD start sites (E) and LAD end sites (F)) was plotted. Mild long-range increase in cytosine methylation was observed in S2 compared to S1 at LAD start and end sites. Red line = S2, Blue line = S1. X axis represents genomic location from the centre of the genomic coordinates of the indicated genomic landmarks. Y axis represents methylated cytosine counts in bins with sizes as indicated. Plots were generated using deepTools and coordinates plotted correspond to Hg38 assembly.

Fig S3: A and B: Distribution of methylated cytosines at LINE-1 sequences undergoing GoM (A) and LoM (B) was plotted for 10kb flanks in S1 (blue) and S2 (red). Differential abrupt increases in cytosine methylation was observed as expected at the centre of LINE-1 sequences as these constitute approximately 20% of GoM and LoM regions. Despite difference in levels of cytosine methylation in the centre of LINE-1 sequences, it was consistently lower in S2 throughout the 10kb flanking regions. Thus, the disturbance in methylation caused by CGGBP1 depletion seemed to be occurring differently at the LINE-1 elements than at the flanking regions. Red line = S2, Blue line = S1. X axis represents genomic location from the centre of LINE-1 regions in GoM or LoM regions. Y axis represents methylated cytosine counts in 100 bp bins. LINE-1 coordinates from RepeatMasker output on GoM and LoM sequences were used to extract sequences from Hg38 assembly. Plots were generated using deepTools.

Fig S4: Differential methylation regulation at G4-forming replication origins by CGGBP1. A and B: Distribution of methylated cytosines for S1 and S2 from the centre of origin (10Kb flanks) was plotted. Replication origins occupied by ORC1 maintained low levels of cytosine methylation at the origin centres as compared to the flanking regions (A). However, a subset of ORC1 peaks that forms G4 structure showed relative higher cytosine methylation at the centre upon CGGBP1 depletion (B). C and D: Similarly, replication origins occupied by PHIP showed relative decrease in the cytosine methylation at the centre (C) as compared to that of flanking regions. In contrast, there was significant increase in methylated cytosine at the centre of G4-forming PHIP origins upon CGGBP1 depletion (D). Red line = S2, Blue line = S1. X axis represents genomic location from the centre of ORC1 and PHIP ChIP seq peaks. Y axis represents methylated cytosine counts in 100 bp bins.

Fig S5: Distribution of strand specific cytosine methylation at promoters with GC-skew shows a consistent increase in methylation only upstream of TSS. In presence of CGGBP1, a positive gradient of cytosine methylation from 1kb upstream to 1kb downstream is observed. Upon CGGBP1 depletion this gradient is disturbed giving rise to a mild but very consistent region of methylation gain immediately upstream of the TSS with respect to direction of transcription. Red line = S2, Blue line = S1. X axis represents genomic location from the centre of GC-skew promoter coordinates. Y axis represents methylated cytosine counts in 50 bp bins.

Fig S6: R-loop forming regions undergoing methylation changes are not randomly distributed with respect to TSS. Both at permissive and robust TSSs the GoM and LoM R-loops were clustered within 1kb flanks of the TSS. Frequency plots of minimum distance between GoM/LoM R-loops and closest TSS shows relative abundance of methylation changes at R-loops within 1kb flanks of TSSs. The Y axes show percentages of total TSSs within a bin. X axes show the bins in units of 1000 bases relative to the TSS as zero.

Fig S7: CGGBP1 regulates methylation at cytosine methylation regulatory genes. For all the known TSSs of these genes, the number of methylated cytosine on each strand was counted in 1Kb upstream regions and 1Kb downstream region in absence and presence of CGGBP1. Difference in strand specific methylated cytosine count in absence and presence of CGGBP1 (S2-S1) was plotted for each TSS. Up = upstream 1Kb region; Down = downstream 1 Kb region, Grey bars represent top strand and black bars represent bottom strand).

METHODS

Cell culture, shmiR transduction, bisulfite conversion of DNA, library preparation and Illumina sequencing and raw data analysis

The sequences analyzed in this study were obtained by sequencing of libraries described earlier[1]. The source of DNA, methods of DNA extraction, bisulfite conversion (including lambda phage DNA as spike in for calculating conversion efficiency) and library preparation apply as described before. The sequences obtained for this analysis are different from the ones already published[1]. Briefly, normal human foreskin fibroblasts (1064Sk, passage 12), cultured in MEM (SIGMA) supplemented with 10% FCS (SIGMA) and 0.05% Glutamine (SIGMA) were transduced with CGGBP1-targetting or non-targeting lentiviral shmiRs (Dharmacon Thermoscientific; V3LHS_351828, V3LHS_351826 and V3LHS_351824 for specific and RHS4348 for non-targeting constructs). 96 h post-tranduction, CGGBP1 knock-down was confirmed by western blot and genomic DNA was extracted. After bisulfite conversion (EZ methylation Kit, Zymo Research), library was prepared by using NEXTflex Bisulfite (Boo Scientific (5119–01)) as per manufacturer's protocol. Per 1 microgram DNA, 500 pg Lambda DNA spike (500 pg/ul) to be used as a spike-in control. Paired sequence reads (Illumina) were unpaired before mapping. After trimming of adaptor sequences and filtering out of sequences with ambiguous nucleotides (N), the mapping was performed against unmasked Hg38 using Bismark (version 0.16.2) with inbuilt Bowtie2. The mapping parameters were: "-f --score-min L,0,-0.2 --ignore-quals". Subsequently, Bismark methylextractor was used to extract context and strand specific methylation data for cytosines with specified base locations. In both samples the Lambda DNA spike in controls indicated 95% conversion efficiency.

Nucleotide Composition analyses

For nucleotide composition analyses of the entire sequence data, sequences of all reads (pre-mapping) were used. For nucleotide composition analyses of the mapped sequence data, sequences of all the uniquely mappable reads were used. The data for unmapped reads were derived by subtracting the values for mapped reads from the total reads. Nucleotide compositions were obtained using the EMBOSS tools Compseq and NucBed.

Analyses of genomic coordinates and sequences of regions

GoM, LoM, RoM and RuN locations were obtained by using Bedtools intersect tool on the Bismark methylextractor output. Bedtools merge tool was used to generate regions out of single base coordinates for analyses at the level of regions and their sequences. The parameters of merging are detailed in figure S2. Sequences of thus obtained bed coordinates were extracted from Hg38 (repeat- masked or unmasked as described) using Bedtools getfasta tool. To identify the number of

cytosines (methylated or unmethylated as described) in any bed coordinate, Bedtools coverage tool was used with coverage count option. The distance between two different sets of bed coordinates were obtained using Bedtools Closest tool. For methylation analyses at region level, we retained only the cytosines exhibiting change of methylation only between S1 and S2 (consistently methylated or unmethylated) and filtered out those exhibiting opposite methylation states within a sample (inconsistently methylated).

Repeat content analyses

The repeat-masked and unmasked genome Hg38 were used as available from UCSC genome browser. The subsets of sequence data were repeat-masked using RepeatMasker[2]. The repeat search engine used was RMBLAST (NCBI) and the repeat database used was obtained from RepBase[2,3].

Motif finding

Motif search was done using locally installed versions of MEME (version 4.11.2)[4] suite tools dreme and fimo. Unless described, the motif searched were performed using default options with changes in -k value ranging from 5 to 14.

G4 quadruplex prediction

G4 quadruplex prediction in replication origin peaks was performed using QGRS tool[5]. For identification of G4 forming sequences in larger datasets, the sequence signature {G(3)N(3-5)G(3)N(3-5)G(3)} (and various other combinations thereof) were searched using Fuzznuc (EMBOSS).

GC skew

GC skew was calculated using the formula {(G-C)/(G+C)}[4,6]. The calculations were performed using either inbuilt functions of OpenOffice Spreadsheet or using shell commands of UNIX.

Plotting of signals in genomic coordinates

Methylation signals from S1 and S2 were plotted along genomic coordinates using deepTools[7]. Bed to BigWig conversion was done using UCSC scripts bedItemOverlapCount, bedGraphToBigWig. Plots were generated using plotProfile function on the matrix generated using computeMatrix function.

Statistical analyses

All statistical tests were performed using Prism 7 (GraphPad) on numerical data generated from the above mentioned tools including OpenOffice Spreadsheet.

Publicly available data usage

Following publicly available datasets were used in these analyses: Replication origins[8–10], CTCF-binding sites[11], Enhancers and TSSs (FANTOM database Riken and Ensembl BioMart), CpG islands (UCSC), LADs[12], TADs[13], LINE-1 (Repbase), GC skew regions, GC skew promoters, R-loops genome-wide[14,15], Cytobands (G- bands and R-bands) (UCSC). Coordinates and sequences switched between different Hg assemblies were done using UCSC Liftover tool.

Supplementary References

1. Agarwal, P. et al. CGGBP1 mitigates cytosine methylation at repetitive DNA sequences. BMC Genomics 16, 390 (2015).

2. Website. Available at: A.F.A. Smit, R. Hubley & P. Green RepeatMasker at http://repeatmasker.org. (Accessed: 10th May 2017)

3. Jurka, J. Repbase Update: a database and an electronic journal of repetitive elements. Trends

Genet. 16, 418–420 (2000).

4. Bailey, T. L. et al. MEME SUITE: tools for motif discovery and

searching. Nucleic Acids Res. 37, W202–8 (2009).

5. Kikin, O., D'Antonio, L. & Bagga, P. S. QGRS Mapper: a web- based server for predicting G-quadruplexes in nucleotide sequences. Nucleic Acids Res. 34, W676–82 (2006).

6. Touchon, M., Nicolay, S., Arneodo, A., d'Aubenton-Carafa, Y. & Thermes, C. Transcription-coupled TA and GC strand asymmetries in the human genome. FEBS Lett. 555, 579–582 (2003).

7. Ramírez, F. et al. deepTools2: a next generation web server for deep-sequencing data analysis. Nucleic Acids Res. 44, W160–5 (2016).

8. Dellino, G. I. et al. Genome-wide mapping of human DNA- replication origins: levels of transcription at ORC1 sites regulate origin selection and replication timing. Genome Res. 23, 1–11 (2013).

9. Zhang, Y. et al. A replicator-specific binding protein essential for site-specific initiation of DNA replication in mammalian cells. Nat. Commun. 7, 11748 (2016).

10. Wang, Y. et al. Temporal association of ORCA/LRWD1 to late- firing origins during G1 dictates heterochromatin replication and organization. Nucleic Acids Res. 45, 2490–2502 (2017).

11. Schmidt, D. et al. Waves of retrotransposon expansion remodel genome organization and CTCF binding in multiple mammalian lineages. Cell 148, 335–348 (2012).

12. Guelen, L. et al. Domain organization of human chromosomes revealed by mapping of nuclear lamina interactions. Nature 453, 948– 951 (2008).

13. Dixon, J. R. et al. Topological domains in mammalian genomes identified by analysis of chromatin interactions. Nature 485, 376–380 (2012).

14. Ginno, P. A., Lott, P. L., Christensen, H. C., Korf, I. & Chédin, F. R-loop formation is a distinctive characteristic of unmethylated human CpG island promoters. Mol. Cell 45, 814–825 (2012).

15. Ginno, P. A., Lim, Y. W., Lott, P. L., Korf, I. & Chédin, F. GC skew at the 5' and 3' ends of human genes links R-loop formation to epigenetic regulation and transcription termination. Genome Res. 23, 1590–1600 (2013).

Table S1

	Percent of total sequence data
**A+T in S1**	**64.76**
**G+C in S1**	**35.23**
**A+T in S2**	**63.37**
**G+C in S2**	**36.62**

Table S2

	**S1 total sequenced**	**S1 Hg38 mapped**	**S1 unmapped**	**S2 total sequenced**	**S2 Hg38 mapped**	**S2 unmapped**
**A+T (% of total)**	**64.76**	**96.89**	**66.11**	**63.37**	**96.80**	**64.23**
**G+C (% of total)**	**35.23**	**3.10**	**33.88**	**36.62**	**3.19**	**35.76**

Table S3

**Nucleotide**	**Gain of Methylation**	**Loss of Methylation**
**C (C on OT strand)**	2602632	2538171
**G (C on OB strand)**	2586495	2504526
**N (Repeat-masked C or G)**	10398259	11240514
**Total**	15587386	16283211
**Percentage in repeat**	**66.71**	**69.03**

Table S4

Context	**LoM**	**GoM**	**RuN**	**RoM**	**Total**	**Ratio LoM/ Total**	**Ratio GoM/ Total**
CpG	1814119	1833977	2985768	5389807	12023671	0.1508	0.1525
CHG	2864174	2718468	31726993	795432	38105067	0.0751	0.0713
CHH	11604918	11034941	94340671	5943885	122924415	0.0944	0.0897

Table S5

Context	Loss of methylation	Gain of methylation	Remain Unmethyated	Remain Methyated	Total Methylated	Total Unmethylated	Ratio Met/ Unmet
CpG	1814119	1833977	2985768	5389807	7223784	4799887	1.5049
CHG	2864174	2718468	31726993	795432	3513900	34591167	0.1015
CHH	11604918	11034941	94340671	5943885	16978826	10594558	0.1602

Table S6

	LoM	GoM	RuN	RoM	Total
CpG	1814119	1833977	2985768	5389807	1.202E+07
CHG	2864174	2718468	3.1726E+07	795432	3.810E+07
CHH	1.1604E+07	1.1034E+07	9.4340E+07	5943885	1.2292E+08
**Chi-square test (LoM)**					
Chi-square					142071314
DF					2
P value (two-tailed)					<0.0001
P value summary					****
Is discrepancy significant (P < 0.05)?					Yes
Outcome		Expected #	Observed #	Expected %	Observed %
CpG		12023671	1814119	73.84	11.14
CHG		38105067	2864174	234	17.59
CHH		122924415	11604918	754.9	71.27
TOTAL		173053153	16283211	1063	100.00
**Chi-square test (GoM)**					
Chi-square					143342666
DF					2
P value (two-tailed)					<0.0001
P value summary					****
Is discrepancy significant (P < 0.05)?					Yes
Outcome		Expected #	Observed #	Expected %	Observed %
CpG		12023671	1833977	77.14	11.77
CHG		38105067	2718468	244.5	17.44
CHH		122924415	11034941	788.6	70.79
TOTAL		173053153	15587386	1110	100.00

Table S7

	GoM	LoM
**Percentage composition of repeats**		
**SINEs:**	1.35	1.33
ALUs	**0.85**	**0.89**
MIRs	0.5	0.44
**LINEs:**	19.94	22.43
LINE1	**17.38**	**19.87**
LINE2	2.47	2.48
L3/CR1	0.07	0.08
**LTR elements**	4.89	4.38
ERVL	0.73	0.65
ERVL-MaLRs	1.67	1.59
ERV_classI	2.26	1.97
ERV_classII	0.19	0.14
**DNA elements:**	1.37	1.34
hAT-Charlie	0.33	0.31
TcMar-Tigger	0.78	0.82

Table S8

	**S2 vs S1 methylation status in the flanks**	**S2 vs S1 methylation status in the centre**	**Pattern of methylation distribution**
**CTCF-binding sites**	*Subtle loss of methylation*	*Subtle loss of methylation*	*Enrichment at centre for both S1 and S2*
**Enhancers**	*No change*	*Subtle loss of methylation*	*Depletion at centre for both S1 and S2*
**CpG islands**	*No change*	*Subtle gain of methylation*	*Depletion at centre for both S1 and S2*
**LADs (START)**	*Subtle gain of methylation*	*Subtle gain of methylation*	*No enrichment at LAD start points*
**LADs (END)**	*Subtle gain of methylation*	*Subtle gain of methylation*	*No enrichment at LAD end points*
**TADs (START)**	*No change*	*No change*	*No enrichment at TAD start points*
**TADs (END)**	*No change*	*No change*	*No enrichment at TAD end points*
**LINE-1 elements**	*No change*	*Gain and loss of methylation both at distinct sets of L1 sequences*	*Strong enrichment of methylation events at centre*

Table S9

	Total number of regions	Total combined length of all regions	Number of overlapping regions with GoM (not LoM)	Total combined length of overlapping regions with GoM (not LoM)	Number of overlapping regions with LoM (not GoM)	Total combined length of overlapping regions with LoM (not GoM)
**Robust TSS**	76420	2058560	195	1997	89	916
**Permissive TSS**	217516	3949159	451	3958	217	1899
**Permissive Enhancers**	13388	18334316	769	13388	532	9128
**Universal Enhancers**	200	111112	2	35	10	211
**Insulators**	642820	1056483581	9966	203198	8247	159459
**TADs**	2261	2525209352	5	182	1	14
**LADs**	1296	1133912450	23	887	18	975

Table S10

	GoM (All)	LoM (All)	GoM (L1-free)	LoM (L1-free)	GoM (L1)	LoM (L1)
**Sum of two Gaussians (Best-fit values)**						
**Amplitude1**	8.067	7.988	7.823	7.716	9.705	10.12
**Mean1**	-0.3483	-0.3326	-0.3621	-0.3526	-0.259	-0.22
**SD1**	0.2549	0.2636	0.2631	0.2713	0.2153	0.229
**Amplitude2**	8.261	8.158	8.114	8.023	8.912	8.281
**Mean2**	0.3518	0.3488	0.3655	0.3632	0.2675	0.2929
**SD2**	0.2381	0.2365	0.2416	0.242	0.2184	0.2029
**Goodness of Fit**						
**Robust Sum of Squares**	8.779	8.78	8.399	8.071	12.99	17.03
**RSDR**	0.474	0.4742	0.5734	0.5927	0.1493	0.1234
**Number of points**						
**# of X values**	21	21	21	21	21	21
**# Y values analyzed**	21	21	21	21	21	21

Table S11

		**ORC1**		**ORCA (Mid)**		**ORCA (Late)**		**ORCA (Early)**		**PHIP**	
		OB LoM	OT GoM	OB LoM	OT GoM	OB LoM	OT GoM	OB LoM	OT GoM	OB LoM	OT GoM
**CpG**	OT LoM	0.098	0.295	0.097	0.452	0.273	0.547	0.104	0.412	0.025	0.245
	OB GoM	0.275	0.100	0.459	0.068	0.571	0.212	0.509	0.080	0.265	0.018
**CHG**	OT LoM	-0.078	0.050	-0.019	0.187	0.288	0.404	-0.038	0.128	-0.137	-0.042
	OB GoM	0.048	-0.087	0.255	0.043	0.522	0.333	0.241	-0.032	0.064	-0.130
**CHH**	OT LoM	-0.009	0.236	-0.011	0.855	-0.013	0.911	-0.013	0.814	-0.022	0.345
	OB GoM	0.311	-0.036	0.804	-0.010	0.810	-0.015	0.872	-0.013	0.409	-0.049

Figure S1

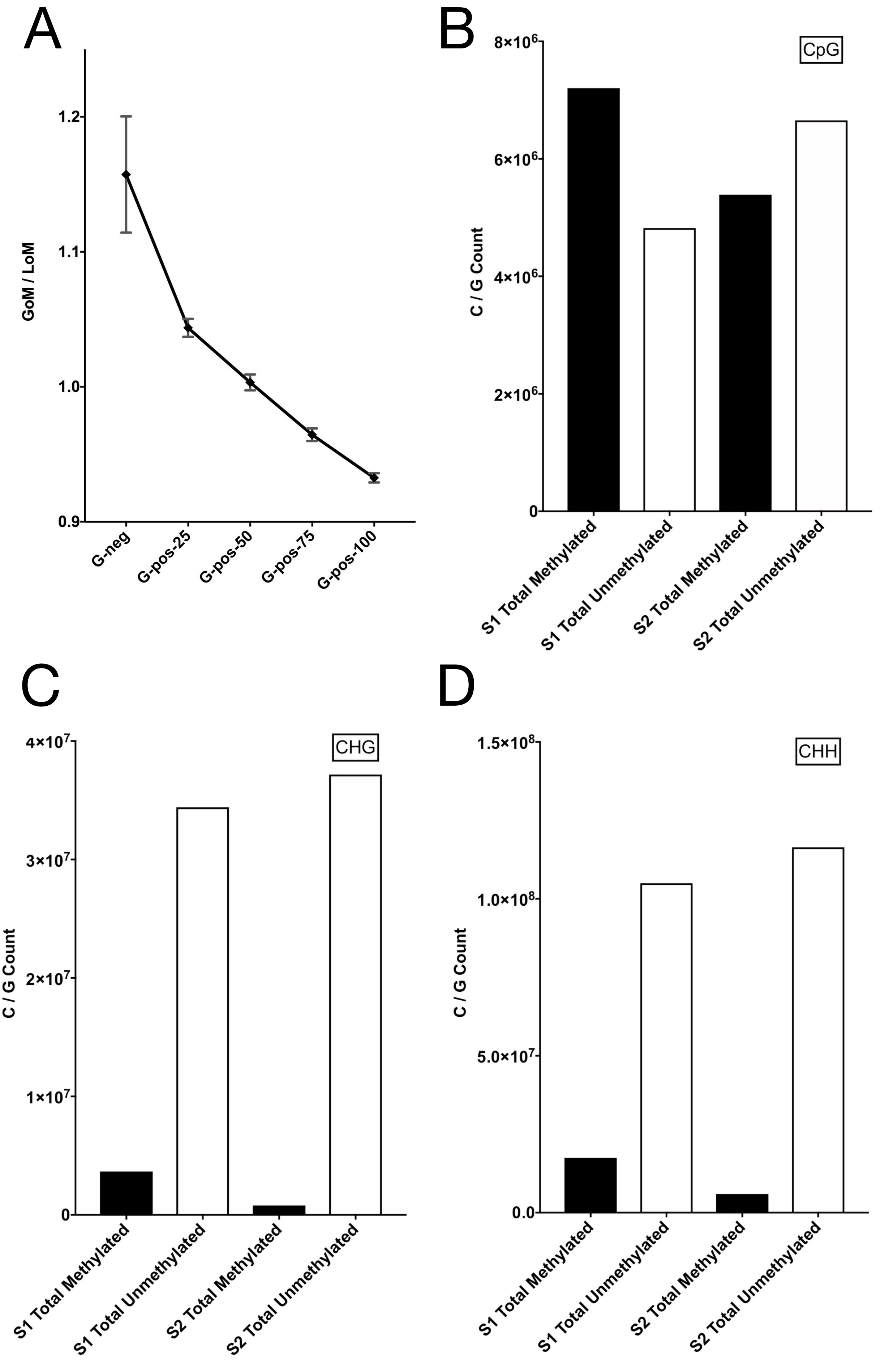
A
GoM / LoM
1.2
1.1
1.0
0.9
G-neg
G-pos-25
G-pos-50
G-pos-75
G-pos-100
B
CpG
C / G Count
8×10^6
6×10^6
4×10^6
2×10^6
0
S1 Total Methylated
S1 Total Unmethylated
S2 Total Methylated
S2 Total Unmethylated
C
CHG
C / G Count
4×10^7
3×10^7
2×10^7
1×10^7
0
S1 Total Methylated
S1 Total Unmethylated
S2 Total Methylated
S2 Total Unmethylated
D
CHH
C / G Count
1.5×10^8
1.0×10^8
5.0×10^7
0.0
S1 Total Methylated
S1 Total Unmethylated
S2 Total Methylated
S2 Total Unmethylated

Figure S2

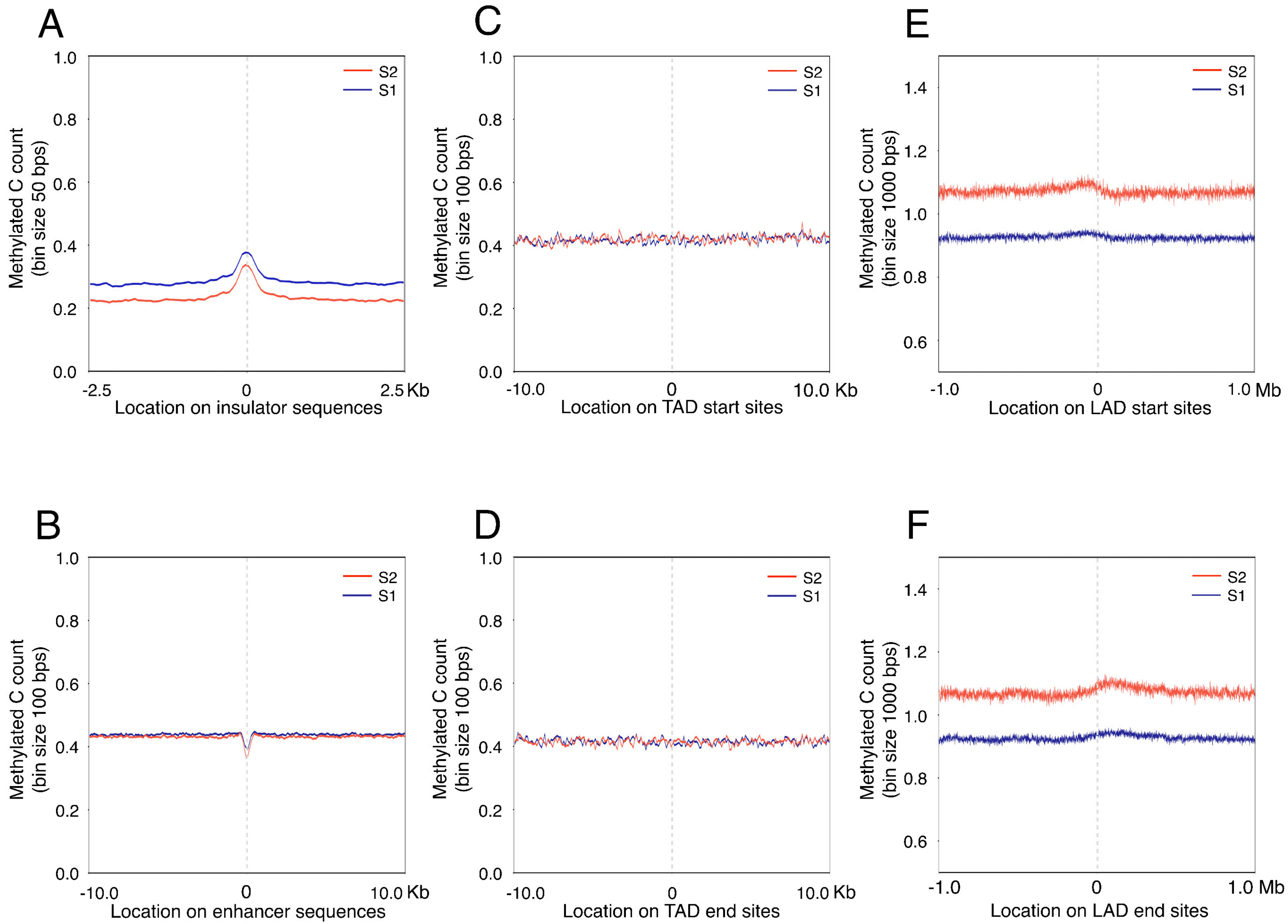
A
S2
S1
Methylated C count (bin size 50 bps)
1.0
0.8
0.6
0.4
0.2
0.0
-2.5
0
2.5 Kb
Location on insulator sequences
B
S2
S1
Methylated C count (bin size 100 bps)
-10.0
0
10.0 Kb
Location on enhancer sequences
C
S2
S1
Methylated C count (bin size 100 bps)
-10.0
0
10.0 Kb
Location on TAD start sites
D
S2
S1
Methylated C count (bin size 100 bps)
-10.0
0
10.0 Kb
Location on TAD end sites
E
S2
S1
Methylated C count (bin size 1000 bps)
1.4
1.2
1.0
0.8
0.6
-1.0
0
1.0 Mb
Location on LAD start sites
F
S2
S1
Methylated C count (bin size 1000 bps)
-1.0
0
1.0 Mb
Location on LAD end sites

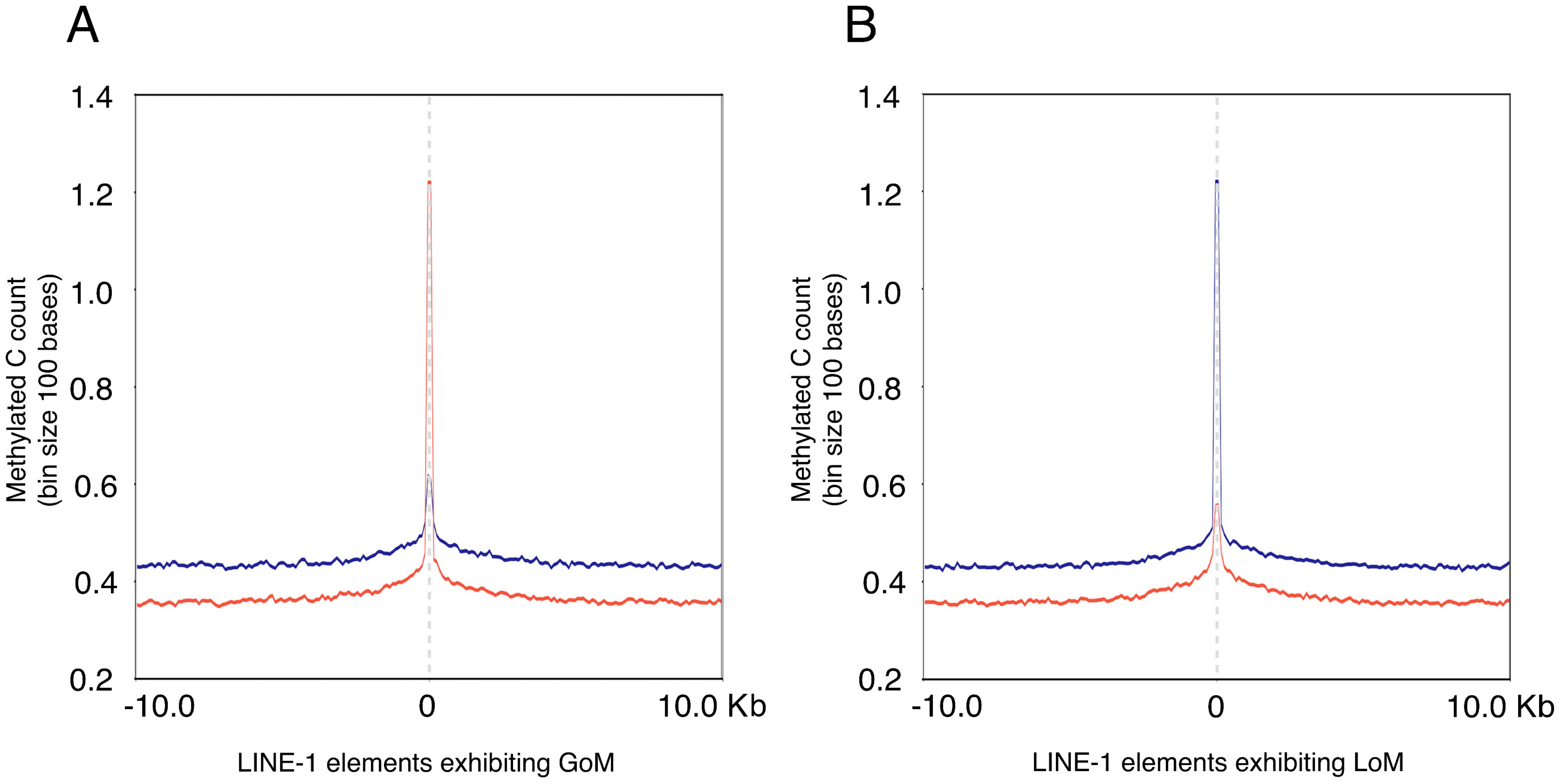
A
B
1.4
1.2
1.0
0.8
0.6
0.4
0.2
Methylated C count
(bin size 100 bases)
-10.0
0
10.0 Kb
LINE-1 elements exhibiting GoM
LINE-1 elements exhibiting LoM

Figure S4

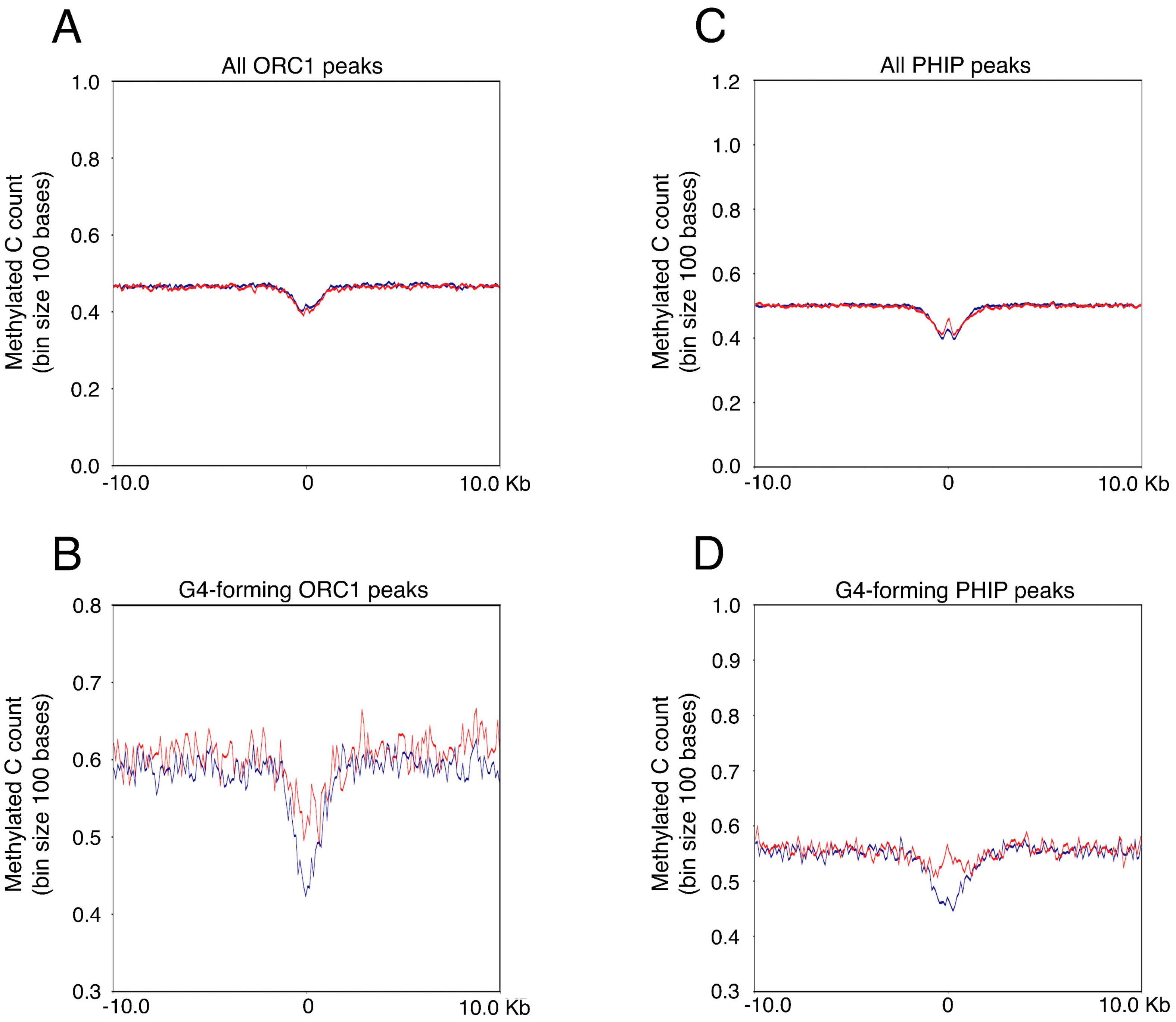
A
All ORC1 peaks
Methylated C count
(bin size 100 bases)
1.0
0.8
0.6
0.4
0.2
0.0
-10.0
0
10.0 Kb
C
All PHIP peaks
Methylated C count
(bin size 100 bases)
1.2
1.0
0.8
0.6
0.4
0.2
0.0
-10.0
0
10.0 Kb
B
G4-forming ORC1 peaks
Methylated C count
(bin size 100 bases)
0.8
0.7
0.6
0.5
0.4
0.3
-10.0
0
10.0 Kb
D
G4-forming PHIP peaks
Methylated C count
(bin size 100 bases)
1.0
0.9
0.8
0.7
0.6
0.5
0.4
0.3
-10.0
0
10.0 Kb

Figure S5

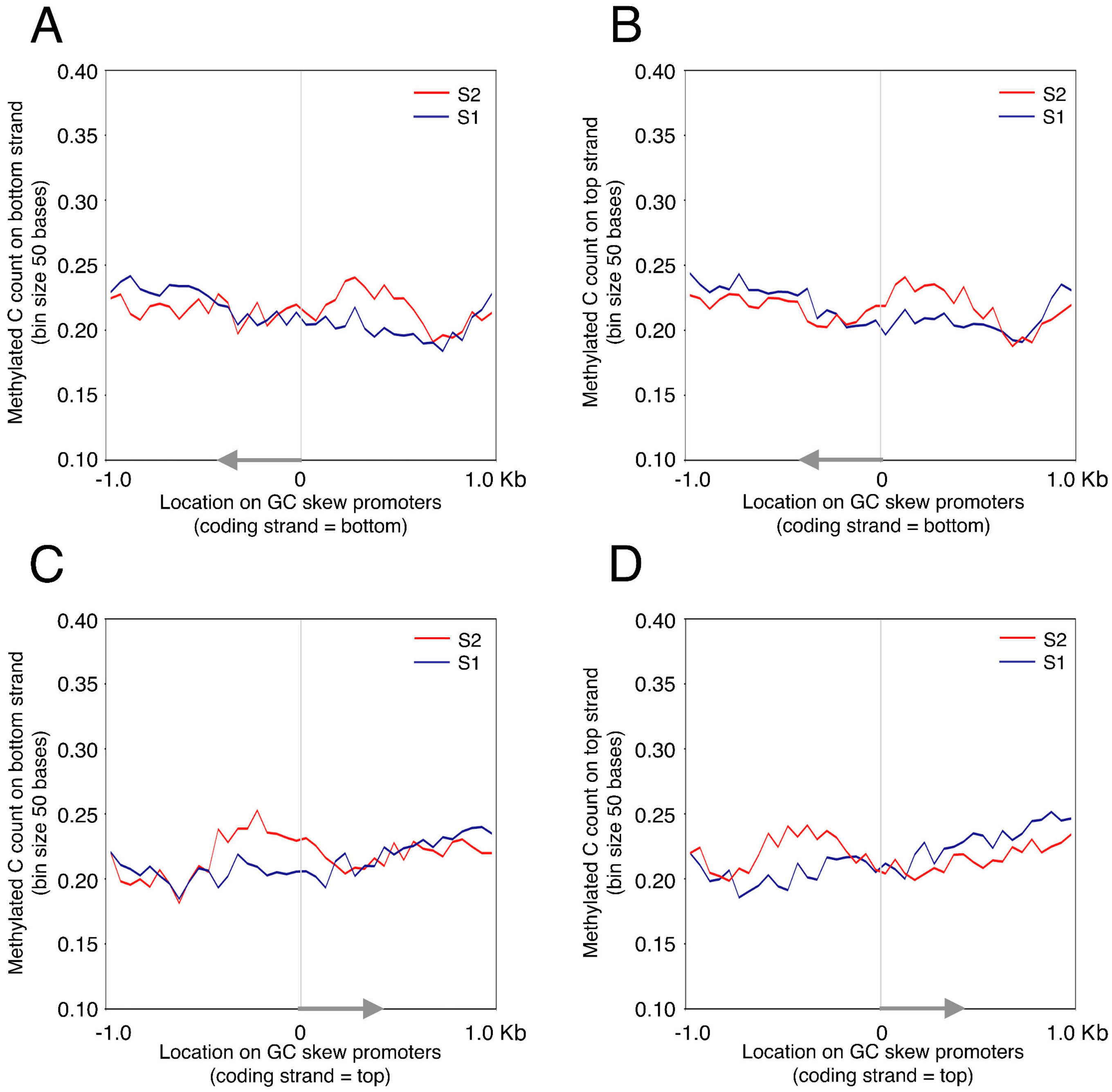
A
B
C
D
Methylated C count on bottom strand
(bin size 50 bases)
Methylated C count on top strand
(bin size 50 bases)
0.40
0.35
0.30
0.25
0.20
0.15
0.10
S2
S1
-1.0
0
1.0 Kb
Location on GC skew promoters
(coding strand = bottom)
Location on GC skew promoters
(coding strand = top)

Figure S6

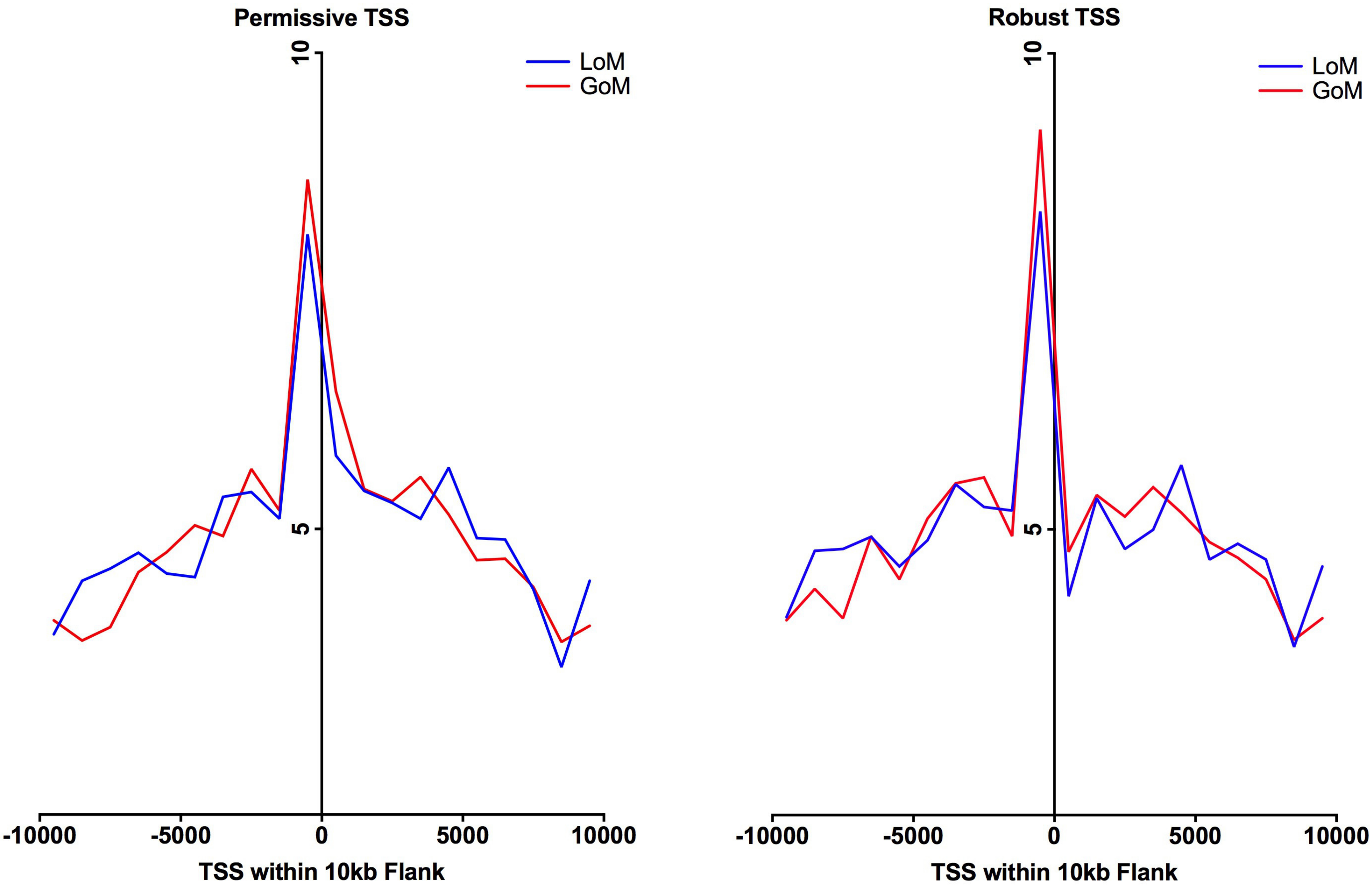
Permissive TSS
LoM
GoM
10
5
-10000
-5000
0
5000
10000
TSS within 10kb Flank
Robust TSS
LoM
GoM
10
5
-10000
-5000
0
5000
10000
TSS within 10kb Flank

Figure S7

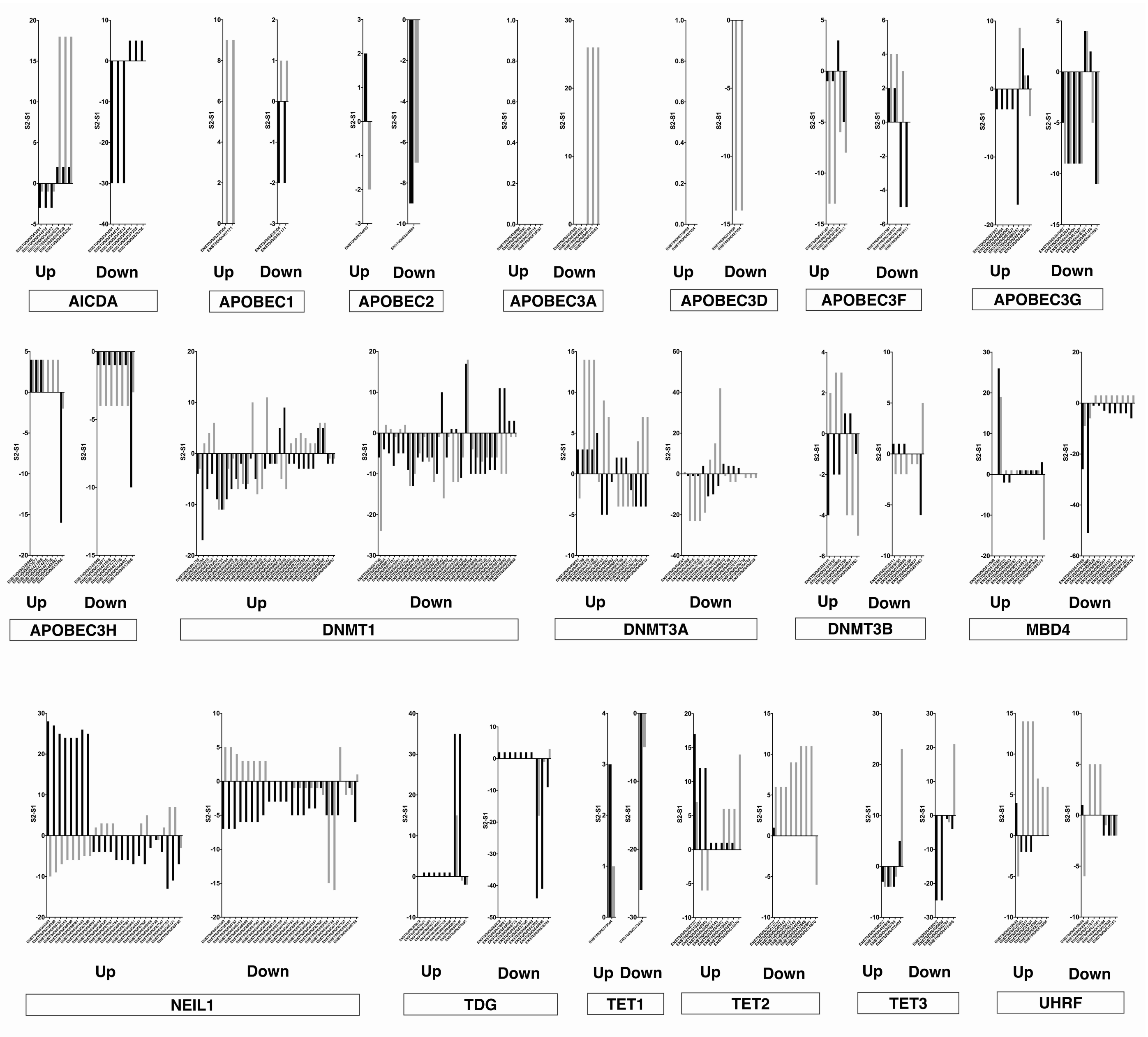
S2-S1
Up
Down
AICDA
APOBEC1
APOBEC2
APOBEC3A
APOBEC3D
APOBEC3F
APOBEC3G
APOBEC3H
DNMT1
DNMT3A
DNMT3B
MBD4
NEIL1
TDG
TET1
TET2
TET3
UHRF
